# Gestational and childhood urinary iodine concentrations and children’s cognitive function in a longitudinal mother-child cohort in rural Bangladesh

**DOI:** 10.1093/ije/dyac110

**Published:** 2022-05-25

**Authors:** Mariza Kampouri, Fahmida Tofail, Syed Moshfiqur Rahman, Klara Gustin, Marie Vahter, Maria Kippler

**Affiliations:** Institute of Environmental Medicine, Karolinska Institutet, Stockholm, Sweden; Maternal and Child Health Division, International Centre for Diarrhoeal Disease Research, Dhaka, Bangladesh; Maternal and Child Health Division, International Centre for Diarrhoeal Disease Research, Dhaka, Bangladesh; Department of Women’s and Children’s Health, Uppsala University, Uppsala, Sweden; Institute of Environmental Medicine, Karolinska Institutet, Stockholm, Sweden; Institute of Environmental Medicine, Karolinska Institutet, Stockholm, Sweden; Institute of Environmental Medicine, Karolinska Institutet, Stockholm, Sweden

**Keywords:** Iodine, gestational iodine, childhood iodine, child development, cognitive development

## Abstract

**Background:**

Severe iodine deficiency adversely affects neurodevelopment; however, evidence regarding the association of non-severe deficiency and child cognitive functioning is inconclusive.

**Methods:**

This prospective mother-child cohort study was nested in a population-based nutritional supplementation trial in Bangladesh (Maternal and Infant Nutrition Interventions in Matlab [MINIMat]). Participants with data on cognitive abilities at 5 and 10 years of age (*n *=* *1530) and at least one measurement of urinary iodine concentration (UIC) (gestational week 8, 5, and 10 years) were selected. Cognitive abilities were assessed using the Wechsler Preschool and Primary Scale of Intelligence (WPPSI-III) and Wechsler Intelligence Scale for Children (WISC-IV). UICs were measured with inductively coupled plasma mass spectrometry and thereafter adjusted for specific gravity.

**Results:**

Median UICs in our population: (282 μg/L [pregnancy]; 406 μg/L [5 years]; 294 μg/L [10 years]) indicated that iodine intake corresponded to above ‘adequate’ or even ‘excessive’, according to the WHO classification. Maternal ‘UIC <150 μg/L’ was associated with lower full-scale and verbal scores at 5 and 10 years, although the associations were weakened in the fully adjusted models. A tendency of decreased verbal scores was also observed for maternal ‘UIC ≥500 μg/L’ but not for the corresponding child iodine category (≥300 μg/L). Child ‘UIC <100 μg/L’ was associated with lower processing speed (B=-3.1, 95% CI [-6.2, -0.1]; *P*-value = 0.041) compared with the reference group (100 μg/L≤ UIC <300 μg/L).

**Conclusions:**

Current findings add to the growing evidence of a causal association of early-life iodine intake with cognitive development, indicating that low iodine intake during childhood is associated with reduced processing speed and non-optimal gestational iodine intake is weakly associated with slightly poorer verbal development outcomes.

Key Messages

- Iodine intake was generally above ‘adequate’ in the rural area of Matlab, Bangladesh.
- Low child urinary iodine concentrations were linked to reduced processing speed at 10 years of age.
- Children’s verbal cognitive development appeared to be susceptible to non-optimal maternal iodine intake during gestation, although the estimates were small.
- Children appeared to be less susceptible to excess iodine intake during childhood than to maternal excessive iodine intake during gestation.

## Introduction

Iodine is an essential micronutrient required for thyroid hormones’ synthesis, and therefore adequate dietary intake is crucial for metabolic control, growth, and development.^1^ In severely iodine-deficient populations, thyroid disorders are highly prevalent and linked with suboptimal fetal neurodevelopment and subsequent irreversible cognitive impairments.^2^^,^^3^ However, the impact of less severe iodine deficiency on children’s cognitive development remains unclear. Insufficient maternal iodine intake during pregnancy, defined as urinary iodine concentration (UIC) <150 μg/L, has been associated with decreased verbal intelligence in a British cohort,^4^ and decreased general cognitive development in a Spanish cohort (defined as UIC <100 μg/L in the latter study).^5^ In a cohort study in the Netherlands, no association was identified between insufficient maternal iodine intake (UIC <150 μg/L) and non-verbal intelligence quotient (IQ) or language comprehension at 6 years of age, despite the previously identified alternations in children’s executive functioning at 4 years.^6^^,^^7^ In a meta-analysis including individual data from the aforementioned British, Spanish and Dutch studies, maternal iodine intake during pregnancy was positively associated with children’s verbal IQ but not with non-verbal cognitive abilities.^8^ In a recent British cohort study there was no consistent evidence of any association between maternal UIC and children’s cognitive function.^9^

Although neurodevelopment continues long after birth, the role of iodine intake during childhood in cognitive development has been understudied. Meta-analyses have estimated a mean intelligence reduction of 12–13.5 points in children living in regions with endemic iodine deficiency.^10^^,^^11^ However, the studies included in the meta-analyses were cross-sectional comparisons between communities with different iodine intakes, and therefore it is impossible to distinguish between the contributions of childhood and gestational deficiency in the reported estimates.^12^ An intervention trial in a region with moderate iodine deficiency has supported the importance of childhood iodine sufficiency, identifying improved cognitive abilities in school-aged children with repleted iodine.^13^

The aim of this study was to explore the impact of early-life iodine intake, assessed through urinary iodine concentrations prenatally and during childhood, on children’s cognitive function at 5 and 10 years of age in a cohort study in rural Bangladesh, an iodine-deficient region where a mandatory salt iodization programme has been in action since 1989^14^ and a wide range of iodine intake has been documented.^15^

## Methods

### Study population

This study is part of a follow-up of a child development cohort nested in a randomized population-based food and micronutrient supplementation trial (Maternal and Infant Nutrition Interventions in Matlab [MINIMat]) during pregnancy in Matlab, a rural sub-district about 50 km southeast from Dhaka, Bangladesh.^16–21^

**Figure 1:**
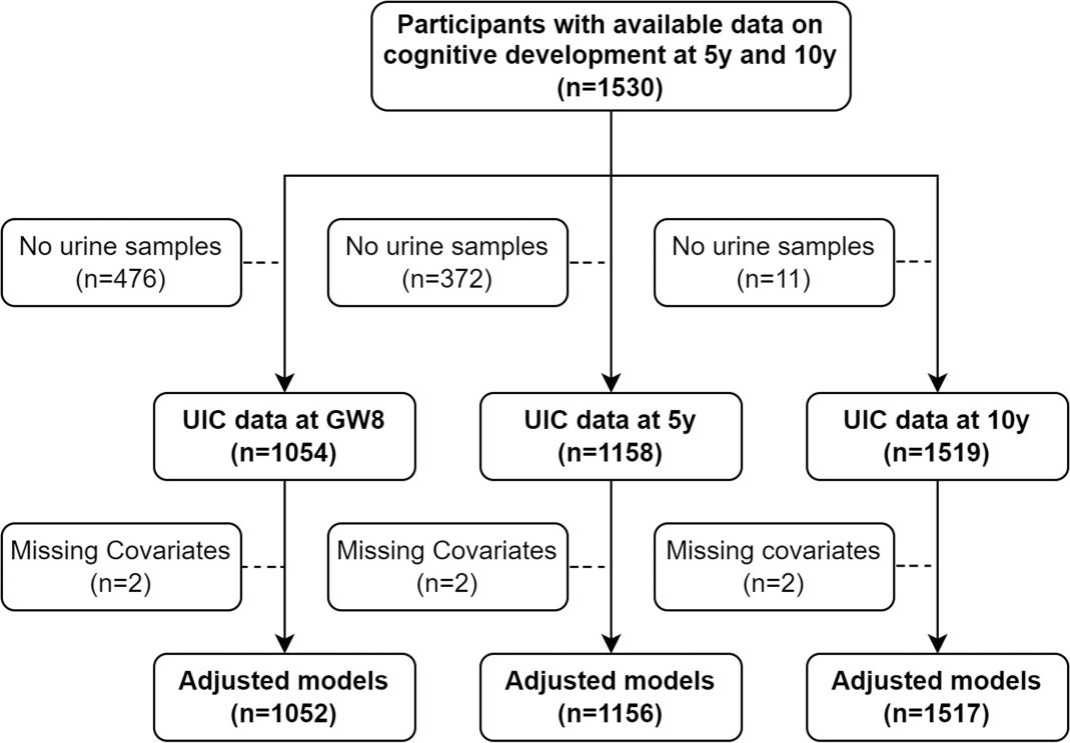
Flow chart for participation in the present study. UIC, urinary iodine concentration; GW8, gestational week 8; 5 y, 5 years; 10 y, 10 years

Initially, infants born in the MINIMat trial between May 2002 and December 2003 (*n* = 2853; twin births excluded) were invited to the child development project, and more than 2000 children were tested at 7 months and 1.5 and 5 years of age.^16^^,^^22^^,^^23^ For the follow-up at 10 years of age, we invited children who were born between October 2002 and December 2003 and were alive and residents in the study area. Of the 1607 invited families, 1530 participated; loss to follow-up (4.8%) was mainly due to out-migration and refusal.^17^^,^^19–21^ We had available data on UICs at 10 years for 1519 participants, at 5 years for 1158 participants, and at gestational week (GW) 8 for 1054 participants (1517, 1156, and 1052 with complete covariate data, respectively) (Figure 1). Missing exposure data were mainly a consequence of unmeasured UICs due to technical issues. Comparison between participants with and without available UIC measurements did not indicate any biologically relevant differences regarding maternal background characteristics and child cognitive scores (Supplementary Table S1, available as Supplementary data at *IJE* online).

### Exposure assessment

Iodine intake was assessed through UIC, a valid biomarker of intake on a population basis.^24^ Spot urine samples were collected at early pregnancy (on average GW8)^25^ and at 5 and 10 years.^21^ The samples were transported in cold-boxes to Matlab Hospital for storage at −70^o^C; then they were transported frozen to Karolinska Institutet, Sweden, for toxic and essential element analyses.

UICs were measured with inductively coupled plasma mass spectrometry (ICP-MS; Agilent 7500ce and 7700x; Agilent Technologies, Tokyo, Japan).^15^ The limit of detection (LOD; SD of the standard blanks * three) for the measurements of iodine in maternal urine and child urine at 5 and 10 years was 0.6 µg/L, 0.1 µg/L, and 0.2 µg/L, respectively. No sample had a UIC below its respective LOD. The analytical quality was ensured by inclusion of two different commercial reference materials in each run (Supplementary Table S2, available as Supplementary data at *IJE* online). The urinary concentrations were adjusted for specific gravity (SG), measured with a digital refractometer (EUROMEX RD712 Clinical Refractometer, Holland), to compensate for dilution variation [UIC*(meanSG-1/individualSG-1)].^26^ We have also measured the concentrations of selenium, arsenic, and cadmium in maternal erythrocytes at GW14^21^^,^^27^ and in urine at 5 and 10 years.^19^^,^^21^^,^^28^

### Outcome assessment

Cognitive development was assessed using the Wechsler Preschool and Primary Scale of Intelligence-Third Edition (WPPSI-III) at 5 years, which includes a verbal, a performance, and a full-scale index,^29^ and the Wechsler Intelligence Scale for Children-Fourth Edition (WISC-IV) at 10 years, which includes a verbal comprehension, a perceptual reasoning, a working memory, a processing speed, and a full-scale index.^30^ Details have been previously published.^16–21^ Both tests were slightly modified to be culturally adapted to the population and testers were trained to administer the tests. WPPSI-III-testers were rotated across the four health care facilities to minimize tester-related bias, and supervisors rated 10% of all tests (interobserver reliability kappa >0.92). Interrater reliability was measured between testers and trainer for WISC-IV, and the training continued until agreement >85% was achieved. The raw scores were used to avoid systematic differences between the present population and the populations used for standardization.

### Potential covariates

Information on maternal background characteristics (age, height, weight, delivery date, education, parity) was collected at the enrolment in MINIMat. Family socioeconomic status (SES) index was derived from information regarding housing and assets ownership, which were obtained during pregnancy and updated at the 10-year assessment. Information on maternal education was collected at the 5-year assessment and updated at the 10-year assessment. Maternal non-verbal reasoning was assessed at the 5-year follow-up (combined Raven’s Standard and Coloured Matrices [RSPM & RCPM]). A modified Home Observation for Measurement Environment questionnaire (HOME) was administered at 5 and 10 years to assess quality of stimulation at home.^21^^,^^31^ Anthropometric measures at birth and at 5 and 10 years were assessed using standard methods and protocols.^32^ Age- and gender-specific z-scores of weight, height and body mass index were estimated according to World Health Organization (WHO) growth standards.^33^ Information on school type was collected at 5 years (none, primary, kindergarten, Madrasa[Islamic], Maktab, non-formal-other) and updated at 10 years (none, primary, English-medium[private], Madrasa, non-profit[NGO]). Children who attended non-formal schools at 5 years were grouped together (Maktab, Madrasa and other-non-formal) and children who did not attend school at 10 years (*n* = 9) were grouped with those who attended Madrasa and non-governmental organization (NGO) schools. Information on schooling duration was collected at 10 years of age.

### Statistical analysis

All statistical analyses were conducted using Stata (version 13, StataCorp LLC, College Station, TX, USA). Bivariate associations were assessed using Spearman’s rank correlation coefficient, Kruskal–Wallis (and Dunn’s test for post-hoc comparisons), Mann–Whitney, chi square, and Fisher’s exact test, depending on the data.

Directed acyclic graphs (DAGs) were constructed to identify the potential confounders based on the background knowledge and using DAGitty version 3.0, (Supplementary Figures S1 and S2, available as Supplementary data at *IJE* online).^34^ Model 1 was adjusted for child gender and the tester (the eight testers at the 5-year follow-up were grouped into three categories, as were the five testers at the 10-year follow-up into three, due to the low number of children assessed by some of the testers) which were a priori selected adjustments. Model 2 was additionally adjusted for socioeconomic status (SES) at enrolment or at 10 years (continuous; assets score), maternal body mass index (BMI) at enrolment (continuous; kg/m^2^), parity at enrolment (continuous; number of children), maternal age at enrolment (continuous; years; in models of child iodine exposure exclusively), maternal non-verbal reasoning measured at 5 years (continuous; raw score; in models of child iodine exposure exclusively) and maternal education at 5 or 10 years (continuous; years).

UICs were not normally distributed, and therefore they were logarithmically transformed (log_2_). Generalized additive models (GAMs; adjusted for all potential confounders) were applied to explore the shape of the relationships between UICs and outcomes (Supplementary Figures S3–S7, available as Supplementary data at *IJE* online; *P-*gain <0.1).^35^ Evidence of non-linearity was identified; thus UICs were categorized using the criteria developed by the WHO for the definition of population iodine intake based on UIC-median,^36^ i.e. UIC <150 μg/L and UIC <100 μg/L indicating insufficient iodine intake for pregnancy and childhood, respectively; 150 μg/L≤ UIC <500 μg/L and 100 μg/L≤ UIC <300 μg/L indicating adequate intake for pregnancy and childhood, respectively; and UIC ≥500 μg/L and UIC ≥300 μg/L indicating excess iodine intake for pregnancy and childhood, respectively. Multivariable-adjusted regression models were applied to assess the associations of interest. The groups indicating iodine adequacy were used as the reference groups in the models. We have used a robust-standard-errors option in the regression models to correct for heteroscedasticity of the errors’ variance. We did not observe any indication of multicollinearity (criteria: pairwise correlations <0.8, and variance inflation factor [VIF] <5).

Sensitivity analyses were conducted with further adjustments for erythrocyte selenium, arsenic, and cadmium concentrations at GW14 or for urinary selenium, cadmium, and arsenic concentrations at 5 or 10 years. We have, also, adjusted the models for the micronutrient supplementation intervention, which was initiated at gestational week 14 (6 weeks after the UICs measurement) and included three interventional groups [(i) 60 mg iron and 400 μg folic acid, (ii) 30 mg iron and 400 μg folic acid and (iii) 15 micronutrients including 150 μg iodine].

## Results

### Background characteristics

The participants’ characteristics are presented in Table 1. Median UIC corresponded to ‘above required’ intake at GW8 (282 μg/L; *n *=* *1054), and to ‘excessive’ (406 μg/L; *n *=* *1157) and ‘above required’ intake (294 μg/L; *n *=* *1519) at 5 and 10 years, respectively.^36^ UICs at the three time points were correlated to each other (rho = 0.27–0.40; *P *<0.001) and positively associated with multiple cognitive abilities’ scores (Table 2).

**Table 1:** Main characteristics of the participating mother-child pairs (*n* = 1530)

	*n*	Mean (±SD) or %^a^
Maternal characteristics		
Age at enrolment (GW8; years)	1530	26.5 (6.0)
BMI at enrolment (GW8; kg/m^2^)	1528	20.0 (2.6)
Parity at enrolment (GW8)	1530	1.5 (1.4)
SES at enrolment (GW8)	1530	−0.2 (2.3)
Micronutrient supplementation group (%)	1530	
Fe30Fol	508	33
Fe60Fol	521	34
MMS (including 150 μg iodine)	501	33
Maternal education at 5 years (years)	1530	4.6 (4.0)
Maternal education at 10 years (years)	1530	5.1 (3.7)
Maternal Raven’s score	1530	25.0 (11.7)
UIC [μg/L; GW8; median (range)]^b^	1054	282 (8, 10 211)
Paternal education at 5 years (years)	1518	5.0 (4.4)
Paternal education at 10 years (years)	1530	5.4 (4.3)
Children’s characteristics at 5 years		
Age	1530	5.4 (0.1)
Height for age (z-score)	1530	−1.6 (0.9)
BMI for age (z-score)	1530	−1.1 (0.8)
HOME score	1465	8.6 (4.9)
School type (%)	1530	
None	311	20
Primary	356	23
Non-formal (including Maktab and Madrasa)	769	50
Kindergarten	94	6
UIC [μg/L; median (range)]^b^	1157	406 (26, 2905)
Tester (%)	1530	
Tester 1	122	8
Tester 2	1192	78
Tester 3	216	14
WPPSI-III: Full-scale score	1530	79.2 (22.4)
WPPSI-III: Verbal scale score	1530	32.8 (10.8)
WPPSI-III: Performance scale score	1530	34.1 (7.8)
Children’s characteristics at 10 years		
Age (years)	1530	9.5 (0.1)
Height for age (z-score)	1530	−1.4 (0.9)
BMI for age (z-score)	1530	−1.3 (1.1)
HOME score	1530	27.0 (5.0)
Number of years at school	1530	3.0 (1.0)
School type (%)	1530	
Primary	1170	76
English medium	151	10
Other (including none, Madrasa, NGO)	209	14
UIC [μg/L; median (range)]^b^	1519	294 (39, 3392)
Tester (%)	1530	
Tester 1	404	26
Tester 2	379	25
Tester 3	363	24
Tester 4	384	25
WISC-IV: Full-scale score	1530	132.4 (33.1)
WISC-IV: Verbal comprehension score	1530	36.7 (10.5)
WISC-IV: Perceptual reasoning score	1530	31.7 (11.6)
WISC-IV: Working memory score	1530	29.7 (6.1)
WISC-IV: Processing speed score	1530	34.2 (11.7)

UIC, urinary iodine concentration; GW, gestational week; HOME, quality of children’s stimulation at home assessed through modified Home Observation for Measurement of the Environment; NGO, non-governmental organization; SES, socioeconomic status assessed through a wealth index based on family ownership; Fe30Fol: Ferum 30 mg & Folate 400 μg; Fe60Fol: Ferum 60 mg & Folate 400 μg; MMS: Multiple Micronutrient Supplementation (Ferum 30 mg, Folate 400 μg, 13 additional micronutrients including 150 μg of iodine); BMI, body mass index; WPPSI-III, Wechsler Preschool & Primary Scale of Intelligence, Third Edition; WISC-IV, Wechsler Intelligence Scale for Children, Fourth Edition.

a Mean (SD) is reported for continuous variables and % frequency for categorical variables, unless stated otherwise.

b UIC is adjusted for specific gravity.

**Table 2:** Spearman correlations of maternal urinary iodine concentrations at gestational week (GW) 8, child urinary iodine concentrations at 5 years and 10 years, and child cognitive abilities’ scores at 5 and 10 years of age

	UIC at GW8^a^	UIC at 5 years^a^	UIC at 10 years^a^
UIC at 5 years^a^	0.27 (<0.001)	–	–
UIC at 10 years^a^	0.27 (<0.001)	0.40 (<0.001)	–
WPPSI-III: Full-scale score	0.09 (0.005)	0.06 (0.043)	NA
WPPSI-III: Verbal scale score	0.09 (0.004)	0.04 (0.216)	NA
WPPSI-III: Performance scale score	0.05 (0.078)	0.06 (0.059)	NA
WISC-IV: Full-scale score	0.08 (0.010)	0.07 (0.018)	0.04 (0.087)
WISC-IV: Verbal comprehension score	0.08 (0.013)	0.07 (0.017)	0.04 (0.083)
WISC-IV: Perceptual reasoning score	0.05 (0.113)	0.05 (0.070)	0.01 (0.612)
WISC-IV: Working memory score	0.10 (<0.001)	0.09 (0.002)	0.04 (0.144)
WISC-IV: Processing speed score	0.06 (0.069)	0.04 (0.131)	0.05 (0.034)

UIC, urinary iodine concentration; WPPSI-III, Wechsler Preschool & Primary Scale of Intelligence, Third Edition; WISC-IV, Wechsler Intelligence Scale for Children, Fourth Edition; NA, non-applicable.

a UIC is adjusted for specific gravity.

### Maternal UICs and children’s cognitive abilities

In the minimally-adjusted models (Model 1), maternal ‘UIC <150 μg/L’ (group median = 94 μg/L) was associated with lower full-scale scores at 5 and 10 years, compared with the reference groups (Table 3). These associations seemed to be mainly driven by the verbal scales. In the fully-adjusted models (Model 2), the estimates were weakened (Table 3). ‘UIC ≥500 μg/L’ (group median = 847 μg/L) was not associated with any of the outcomes in Model 1, but the estimates of the full-scale and the verbal scale were decreased in Model 2 [verbal scale at 5 years: *B* =-1.0, 95% CI (-2.5, 0.5); *P *=* *0.187; verbal-comprehension scale at 10 years: *B* = -0.6, 95% CI (-1.9, 0.8); *P *=* *0.417]. Model 2 estimates were more sensitive to adjustment for family SES and maternal education than for parity and maternal BMI. Further adjustments for erythrocyte selenium, cadmium and arsenic, and for micronutrient supplementation during pregnancy had marginal impact on the results (Supplementary Table S3, available as Supplementary data at *IJE* online).

**Table 3:** Multivariable linear regression models of maternal urinary iodine concentrations at gestational Week 8, categorized according to World Health Organization (2013) cut-offs for population iodine intake classification, with their children’s cognitive abilities scores at 5 and 10 years of age

	Categories of maternal urinary iodine (µg/L) at gestational Week 8^a^							
	UIC <150 μg/L			150 μg/L≤UIC <500 μg/L		UIC ≥500 μg/L		
	*n*	B (95% CI)	*P*-value	*n*		*n*	B (95% CI)	*P*-value
**Cognition at 5 years: WPPSI-III^b^**								
Full-scale score								
Model 1	277	**−4.6 (−8.0, −1.3)**	0.007	496	ref	279	0.8 (−2.7, 4.3)	0.639
Model 2	277	−1.3 (−4.3, 1.7)	0.401	496	ref	279	−1.0 (−4.0, 2.0)	0.527
Verbal scale score								
Model 1	277	**−2.5 (−4.2, −0.9)**	0.002	496	ref	279	−0.3 (−2.0, 1.4)	0.723
Model 2	277	−1.2 (−2.7, 0.3)	0.124	496	ref	279	−1.0 (−2.5, 0.5)	0.187
Performance scale score								
Model 1	277	−1.0 (−2.2, 0.2)	0.105	496	ref	279	0.5 (−0.7, 1.7)	0.403
Model 2	277	0.0 (−1.1, 1.1)	0.945	496	ref	279	−0.0 (−1.1, 1.0)	0.928
**Cognition at 10 years: WISC-IV^c^**								
Full-scale score								
Model 1	277	**−4.8 (−9.5, −0.0)**	0.049	496	ref	279	1.1 (−4.0, 6.1)	0.680
Model 2	277	0.3 (−3.9, 4.5)	0.882	496	ref	279	−0.0 (−4.3, 4.2)	0.986
Verbal comprehension score								
Model 1	277	**−2.1 (−3.6, −0.7)**	0.004	496	ref	279	−0.3 (−1.9, 1.4)	0.759
Model 2	277	−0.6 (−1.8, 0.7)	0.400	496	ref	279	−0.6 (−1.9, 0.8)	0.417
Perceptual reasoning score								
Model 1	277	−1.0 (−2.7, 0.6)	0.211	496	ref	279	0.6 (−1.2, 2.3)	0.520
Model 2	277	0.3 (−1.2, 1.9)	0.657	496	ref	279	0.3 (−1.2, 1.9)	0.680
Working memory score								
Model 1	277	−0.4 (−1.3, 0.4)	0.333	496	ref	279	0.6 (−0.3, 1.5)	0.173
Model 2	277	0.2 (−0.6, 1.0)	0.631	496	ref	279	0.5 (−0.4, 1.3)	0.286
Processing speed score								
Model 1	277	−1.2 (−2.9, 0.6)	0.186	496	ref	279	0.1 (−1.6, 1.8)	0.892
Model 2	277	0.3 (−1.3, 1.9)	0.695	496	ref	279	−0.3 (−1.8, 1.3)	0.751

UIC, urinary iodine concentration; WPPSI-III, Wechsler Preschool & Primary Scale of Intelligence, Third Edition; WISC-IV, Wechsler Intelligence Scale for Children, Fourth Edition.

Bold indicates a *P* value < 0.05.

a UIC is adjusted for specific gravity.

b Analyses of outcomes at 5 years: Model 1 is adjusted for child gender (categorical; male/female), child age (continuous; years) and the tester of the assessment (categorical; 3 categories); Model 2 is additionally adjusted for maternal body mass index at enrolment (continuous; kg/m^2^), parity (continuous; number of children), family socioeconomic status at enrolment (continuous; assets score) and maternal education at 5 years (continuous; years).

c Analyses of outcomes at 10 years: Model 1 is adjusted for child gender (categorical; male/female), child age (continuous; years) and the tester of the assessment (categorical; 4 categories); Model 2 is additionally adjusted for maternal body mass index at enrolment (continuous; kg/m^2^), parity (continuous; number of children), family socioeconomic status at 10 years (continuous; assets score) and maternal education at 10 years (continuous; years).

### Child UICs at 5 years and cognitive abilities

In the cross-sectional analyses at 5 years (Table 4), ‘UIC <100 µg/L’ (group median = 74 μg/L) was associated with lower performance-scores compared with the reference group, although weakened in the fully-adjusted model. ‘UIC ≥300 μg/L’ (group median = 519) was associated with higher scores in comparison with the reference group [full-scale-Model 2: B = 2.9, 95% CI (0.3, 5.4); *P *=* *0.026]. In the longitudinal analyses (Model 1; Table 4), ‘UIC <100 μg/L’ was associated with lower scores in the full-scale at 10 years compared with the reference group (driven mainly by the processing-speed scale). In Model 2, the association with the processing speed was only slightly attenuated [B=-3.1, 95% CI (-6.2, -0.1); *P *=* *0.041]. ‘UIC ≥300 μg/L’ was not associated with cognitive scores at 10 years. Model 2 estimates were more sensitive to adjustment for family SES, maternal education and maternal Raven’s score than for parity, maternal BMI and maternal age.

**Table 4:** Multivariable linear regression models of child urinary iodine concentrations at 5 years, categorized according to World Health Organization (2013) cut-offs for population iodine intake classification, with cognitive abilities’ scores at 5 and 10 years of age

	Categories of child urinary iodine (µg/L) at 5 years^a^							
	UIC <100 μg/L			100 μg/L≤ UIC <300 μg/L		UIC ≥300 μg/L		
	*n*	B (95% CI)	*P*-value	*n*		*n*	B (95% CI)	*P-*value
**Cognition at 5 years: WPPSI-III^b^**								
Full-scale score								
Model 1	43	−4.0 (−11.0, 2.9)	0.254	327	ref	786	**4.0 (1.1, 6.8)**	0.007
Model 2	43	−0.6 (−6.6, 5.3)	0.832	327	ref	786	**2.9 (0.3, 5.4)**	0.026
Verbal scale score								
Model 1	43	−0.2 (−3.2, 2.8)	0.899	327	ref	786	**1.5 (0.1, 2.9)**	0.035
Model 2	43	1.1 (−1.5, 3.7)	0.402	327	ref	786	1.1 (−0.2, 2.4)	0.100
Performance scale score								
Model 1	43	**−2.8 (−5.2, −0.4)**	0.024	327	ref	786	**1.2 (0.2, 2.2)**	0.022
Model 2	43	−1.8 (−4.0, 0.5)	0.121	327	ref	786	0.9 (−0.1, 1.8)	0.069
**Cognition at 10 years: WISC-IV^c^**								
Full-scale score								
Model 1	43	**−10.6 (−20.3, −0.9)**	0.033	327	ref	786	3.4 (−0.8, 7.7)	0.113
Model 2	43	−5.3 (−12.4, 1.9)	0.148	327	ref	786	1.5 (−2.3, 5.3)	0.433
Verbal comprehension score								
Model 1	43	−1.4 (−4.3, 1.3)	0.309	327	ref	786	1.1 (−0.2, 2.4)	0.108
Model 2	43	0.1 (−2.1, 2.4)	0.905	327	ref	786	0.5 (−0.7, 1.6)	0.434
Perceptual reasoning score								
Model 1	43	**−3.5 (−6.7, −0.3)**	0.034	327	ref	786	1.0 (−0.4, 2.5)	0.172
Model 2	43	−2.0 (−4.6, 0.6)	0.136	327	ref	786	0.4 (−0.9, 1.8)	0.530
Working memory score								
Model 1	43	−1.0 (−3.0, 1.0)	0.332	327	ref	786	0.7 (−0.1, 1.6)	0.079
Model 2	43	−0.3 (−2.0, 1.5)	0.770	327	ref	786	0.5 (−0.2, 1.3)	0.178
Processing speed score								
Model 1	43	**−4.7 (−8.2, −1.1)**	0.011	327	ref	786	0.6 (−0.9, 2.1)	0.423
Model 2	43	**−3.1 (−6.2, −0.1)**	0.041	327	ref	786	0.1 (−1.3, 1.5)	0.905

UIC, urinary iodine concentration; WPPSI-III, Wechsler Preschool & Primary Scale of Intelligence, Third Edition; WISC-IV, Wechsler Intelligence Scale for Children, Fourth Edition.

a UIC is adjusted for specific gravity.

b Analyses of outcomes at 5 years: Model 1 is adjusted for child gender (categorical; male/female), child age (continuous; years) and the tester of the assessment (categorical; 3 categories); Model 2 is additionally adjusted for family socioeconomic status at enrolment (continuous; assets score), maternal body mass index at enrolment (continuous; kg/m^2^), maternal age at enrolment (continuous; years), maternal score at Raven’s test (continuous; raw score), maternal education (continuous; years) and parity (continuous; number of children).

c Analyses of outcomes at 10 years: Model 1 is adjusted for child gender (categorical; male/female), child age (continuous; years) and the tester of the assessment (categorical; 4 categories); Model 2 is additionally adjusted for family socioeconomic status at 10 years (continuous; assets score), maternal body mass index at enrolment (continuous; kg/m^2^), maternal age at enrolment (continuous; years), maternal score at Raven’s test (continuous; raw score), maternal education (continuous; years) and parity (continuous; number of children).

We did not observe any meaningful change of the associations after further adjustment for urinary selenium, cadmium and arsenic at 5 years, nor after further adjustment for micronutrient supplementation during pregnancy (Supplementary Table S4, available as Supplementary data at *IJE* online).

### Child UIC at 10 years and cognitive abilities

In the cross-sectional analyses of cognitive abilities at 10 years (Table 5), ‘UIC <100 µg/L’ (group median = 83 μg/L) was associated with lower processing-speed scores compared with the reference group [Model 2: B=-2.8, 95% CI (-5.3, -0.2); *P *=* *0.033]. None of the cognitive abilities’ scores differed when comparing children in the highest UIC category (group median = 478 μg/L) with those in the reference group. Model 2 estimates were more sensitive to adjustment for family SES, maternal education and maternal Raven’s score than for parity, maternal BMI and maternal age. The results did not markedly change after additional adjustment for urinary selenium, cadmium, and arsenic or for micronutrient supplementation during pregnancy (Supplementary Table S5, available as Supplementary data at *IJE* online).

**Table 5:** Multivariable linear regression models of child urinary iodine concentrations at 10 years, categorized according to World Health Organization (2013) cut-offs for population iodine intake classification, with cognitive abilities’ scores at 10 years of age

	Categories of child urinary iodine (µg/L) at 10 years^a^							
	UIC <100 μg/L			100μg/L≤UIC <300μg/L		UIC ≥300 μg/L		
	*n*	B (95% CI)	*P*-value	*n*		*n*	B (95% CI)	*P*-value
**Cognition at 10 years: WISC-IV^b^**								
Full-scale score								
Model 1	59	−2.9 (−11.7, 5.9)	0.515	712	ref	746	2.1 (−1.3, 5.4)	0.231
Model 2	59	−0.5 (−8.2, 7.2)	0.900	712	ref	746	−1.5 (−4.5, 1.4)	0.303
Verbal comprehension score								
Model 1	59	1.0 (−1.6, 3.7)	0.440	712	ref	746	0.8 (−0.2, 1.9)	0.123
Model 2	59	1.7 (−0.6, 4.1)	0.139	712	ref	746	−0.3 (−1.2, 0.7)	0.559
Perceptual reasoning score								
Model 1	59	−0.5 (−3.9, 2.9)	0.778	712	ref	746	0.4 (−0.8, 1.6)	0.503
Model 2	59	0.2 (−2.9, 3.4)	0.898	712	ref	746	−0.7 (−1.7, 0.4)	0.208
Working memory score								
Model 1	59	0.0 (−1.4, 1.5)	0.984	712	ref	746	0.4 (−0.3, 1.0)	0.246
Model 2	59	0.3 (−1.0, 1.7)	0.622	712	ref	746	−0.1 (−0.6, 0.5)	0.854
Processing speed score								
Model 1	59	**−3.5 (−6.2, −0.7)**	0.013	712	ref	746	0.5 (−0.7, 1.7)	0.459
Model 2	59	**−2.8 (−5.3, −0.2)**	0.033	712	ref	746	−0.5 (−1.6, 0.6)	0.353

UIC, urinary iodine concentration; WISC-IV, Wechsler Intelligence Scale for Children, Fourth Edition.

a UIC is adjusted for specific gravity.

b Analyses of outcomes at 10 years: Model 1 is adjusted for child gender (categorical; male/female), child age (continuous; years) and the tester of the assessment (categorical; 4 categories); Model 2 is additionally adjusted for family socioeconomic status at 10 years (continuous; assets score), maternal body mass index at enrolment (continuous; kg/m^2^), maternal age at enrolment (continuous; years), maternal score at Raven’s test (continuous; raw score), maternal education (continuous; years) and parity (continuous; number of children).

## Discussion

To the best of our knowledge, this study is the first to evaluate child cognitive abilities in relation to iodine intake in Bangladesh. Non-optimal gestational iodine intake measured through urinary iodine concentrations was, non-significantly, associated with adverse verbal outcomes and low child iodine intake was associated with reduced processing speed, although only a few children had iodine concentrations <100 µg/L in their urine. Importantly, the associations were relatively weak and did not support that poor iodine intake is an important factor for impaired cognitive function in children aged 5–10 years in a rural area south east of Dhaka. Bangladesh is a country with known endemic iodine deficiency^37^ and a government-mandatory salt-iodization programme since 1989.^14^ The results support previous findings that demonstrated the success of initiatives to combat iodine deficiency.^38^ In fact, both mothers and children of the MINIMat cohort had ‘above requirements’ iodine intake. However, current findings may not be representative for the entire Bangladesh; indicatively, another study has reported persistent iodine deficiency in the north west of the country.^39^

The lack of strong associations may reflect the general iodine sufficiency in the population. A UIC-median of 282 µg/L was found in the pregnant women, i.e. almost twice the WHO cut-off (150 µg/L).^36^ The median UIC of the children at 5 and 10 years of age was 406 µg/L and 294 µg/L, respectively, i.e. well above the WHO cut-off for adequate iodine nutrition (>100 µg/L).^36^ In fact, the UIC of the 5-year-olds even corresponded to excessive iodine intake.^36^ Current results are in line with findings from the Generation R study in the Netherlands, in which a similar median UIC was reported in pregnant women (297 μg/g creatinine) and no clear association was identified between ‘UIC <150μg/g creatinine’ during early pregnancy and children’s non-verbal cognitive scores or verbal comprehension at 6 years.^7^ In the ALSPAC study in the UK, where the median UIC was markedly lower than that of the current study (119 μg/g creatinine), maternal ‘UIC <150μg/g creatinine’ during early pregnancy was associated with increased risk for suboptimal verbal intelligence.^4^ This supports that the iodine intake of the population, as indicated by the median UIC, is an important factor to consider in the interpretation of study results and the need for mitigations.^40^ Indeed, we have previously found that only 6% of the women in a smaller sample of the MINIMat cohort maintained a UIC of <150 µg/L across pregnancy (UIC measured at GW8, 14, 20, and 31).^15^

Although there was no strong association of maternal UIC with their children’s cognitive abilities in this cohort, the results are in line with previous studies identifying verbal-specific associations of low prenatal iodine intake and children’s cognition.^4^^,^^8^^,^^9^ Moreover, children to mothers with gestational ‘UIC >500 μg/L’ also had slightly lower verbal scores compared with the reference group (similar to the associations identified between low gestational iodine intake and verbal abilities). These findings indicate that verbal cognitive development may be susceptible to suboptimal maternal gestational iodine intake. Previous studies on the link between excessive iodine intake and cognitive development are scarce, although excess iodine may affect thyroid function.^41–43^ Observational studies on the cognitive benefits of iodine supplementation in pregnant women with mild-moderate iodine deficiency have shown equivocal results and have also suggested that excessive iodine intake may be linked to adverse cognitive outcomes.^44^^,^^45^

In our population, ‘UIC <100 μg/L’ at 5 and 10 years of age, although present in only 4% of the children, was associated with lower processing speed at 10 years (about 0.3 SD lower than the reference group), which is in line with findings from a randomized controlled trial which showed that iodine supplementation in school-aged children in a moderately iodine-deficient region benefited fluid intelligence and processing speed.^13^ This association may be explained by structural alterations in brain architecture, since thyroid hormones are involved in myelination,^46^^,^^47^ which continues during childhood and supports processing speed.^48^^,^^49^ Children appeared to be less susceptible to excess iodine intake during childhood than to prenatal excessive iodine, since we found no decrease in cognitive ability scores in children with ‘UIC ≥300 μg/L’.

The large number of participants and the early study enrolment which resulted in early-pregnancy UIC assessment are important strengths of this study. The assessment of UICs both prenatally and during childhood by ICP-MS, the repeated cognition assessment and the adequate confounding adjustment are additional strengths. We did not have information on the use of iodized table salt, and the use of single-spot urine samples is a valid way to determine iodine intake at the population-level only. Nevertheless, UIC seemed to be a fairly reliable biomarker of iodine intake in our population as the UICs at the three time points were correlated to each other. Also, maternal UIC was reasonably constant during gestation (GW8, GW14, GW20, GW31) according to previous findings in a smaller MINIMat-cohort sample.^15^ To minimize the misclassification risk, UICs were adjusted for specific gravity (creatinine unreliable in malnourished populations).^2^ The missing data on UICs at GW8 (*n* = 476) and at 5 years (*n* = 372) reduced the power in the respective models, but the direction and the approximate magnitude of the estimates are not expected to be drastically affected. Last, measurements of thyroid biomarkers were not available in the MINIMat study at the presented follow-ups. In particular, thyroglobulin, a proxy of long-term iodine deficiency, would have been useful for confirmation of the present urinary iodine findings.^50^

## Conclusion

In conclusion, our results support that iodine intake in the Matlab population is ‘above requirements’, both in pregnant women and in children up to 10 years of age. Our findings suggest no major impact of non-optimal maternal iodine intake during pregnancy on cognitive development of their children. Low child iodine intake was, however, associated with reduced processing speed, although only a few children had iodine concentrations <100 µg/L in their urine. On the other hand, a large proportion of the children appeared to have excessive iodine intake but this was not associated with adverse cognitive outcomes. Given the prospective design of our study and the adequate confounding control, we believe that our findings add to the growing evidence that supports a causal association of early-life iodine intake and cognitive development.

## Ethics approval

The study was approved by the research and ethical review committees at icddr, b, Bangladesh (PR-11009) and by the regional Ethical Review Board, Stockholm, Sweden (2012/840–31/1). The study was conducted according to the Declaration of Helsinki.

## Supplementary Material

dyac110_Supplementary_Data

## Data Availability

Data cannot be shared for ethical/privacy reasons.
