# Supplementary material for: Gestational and childhood urinary iodine concentrations and children’s cognitive function in a longitudinal mother-child cohort in rural Bangladesh
Source: Int J Epidemiol. 2022 May 25;52(1):144–55. doi: 10.1093/ije/dyac110 (PMC9908062; doi:10.1093/ije/dyac110)
Supplement: dyac110_Supplementary_Data [file dyac110_supplementary_data.docx]

**Gestational and childhood urinary iodine concentrations and children’s cognitive function in a longitudinal mother-child cohort in rural Bangladesh**

Mariza Kampouri^a^, Fahmida Tofail^b^, Syed Moshfiqur Rahman^b,c^, Klara Gustin^a^, Marie Vahter^a^, Maria Kippler^a^*

^a^Institute of Environmental Medicine, Karolinska Institutet, Stockholm, Sweden.

^b^Maternal and Child Health Division, International Centre for Diarrhoeal Disease Research, Dhaka, Bangladesh.

^c^Department of Women’s and Children’s Health, Uppsala University, Uppsala, Sweden.

**Corresponding author:**

*Maria Kippler, Institute of Environmental Medicine, Karolinska Institutet, Box 210, SE-171 77, Stockholm, Sweden. Telephone: +46 7030303131. Telefax: +46 8 33 69 81. Email: [maria.kippler@ki.se](mailto:maria.kippler@ki.se)

**Table of Contents**

**Supplementary Figure S1.** Directed acyclic graph on the association between maternal iodine intake in pregnancy and child cognitive development 3

**Supplementary Figure S2.** Directed acyclic graph on the association between child iodine intake and cognitive development 4

**Supplementary Figure S3.** Generalized additive models of maternal urinary iodine concentration at gestational week 8 with Wechsler Preschool & Primary Scale of Intelligence, Third Edition 5

**Supplementary Figure S4.** Generalized additive models of maternal urinary iodine concentration at gestational week 8 with Wechsler Intelligence Scale for Children, Fourth Edition 6

**Supplementary Figure S5.** Generalized additive models of maternal urinary iodine concentration at 5 years with Wechsler Preschool & Primary Scale of Intelligence, Third Edition 7

**Supplementary Figure S6.** Generalized additive models of maternal urinary iodine concentration at 5 years with Wechsler Intelligence Scale for Children, Fourth Edition 8

**Supplementary Figure S7**. Generalized additive models of maternal urinary iodine concentration at 10 years with Wechsler Intelligence Scale for Children, Fourth Edition 9

**Supplementary Table S1.** Comparison of maternal background characteristics and cognitive outcomes’

scores between participants with and without available data on iodine intake [Urinary Iodine Intake

(UIC) at pregnancy, 5, and 10 years of age) 10

**Supplementary Table S2.** References values and obtained concentrations of iodine in all reference

materials included in the ICP-MS analyses for quality control 11

**Supplementary Table S3.** Multivariable linear regression models of maternal urinary iodine concentrations at gestational week 8, categorized according to WHO (2013) cut-offs for population iodine-intake classification, with their children’s cognitive abilities scores at 5 and 10 years of age, sensitivity analyses 12

**Supplementary Table S4.** Multivariable linear regression models of child urinary iodine concentrations

at 5 years (categorized according to WHO (2013) cut-offs for population iodine-intake classification)

with cognitive abilities’ scores children at 5 and 10 years of age, sensitivity analyses 14

**Supplementary Table S5.** Multivariable linear regression models of child urinary iodine concentrations

at 10 years (categorized according to WHO (2013) cut-offs for population iodine-intake classification) with cognitive abilities’ scores children at 10 years of age, sensitivity analyses 16


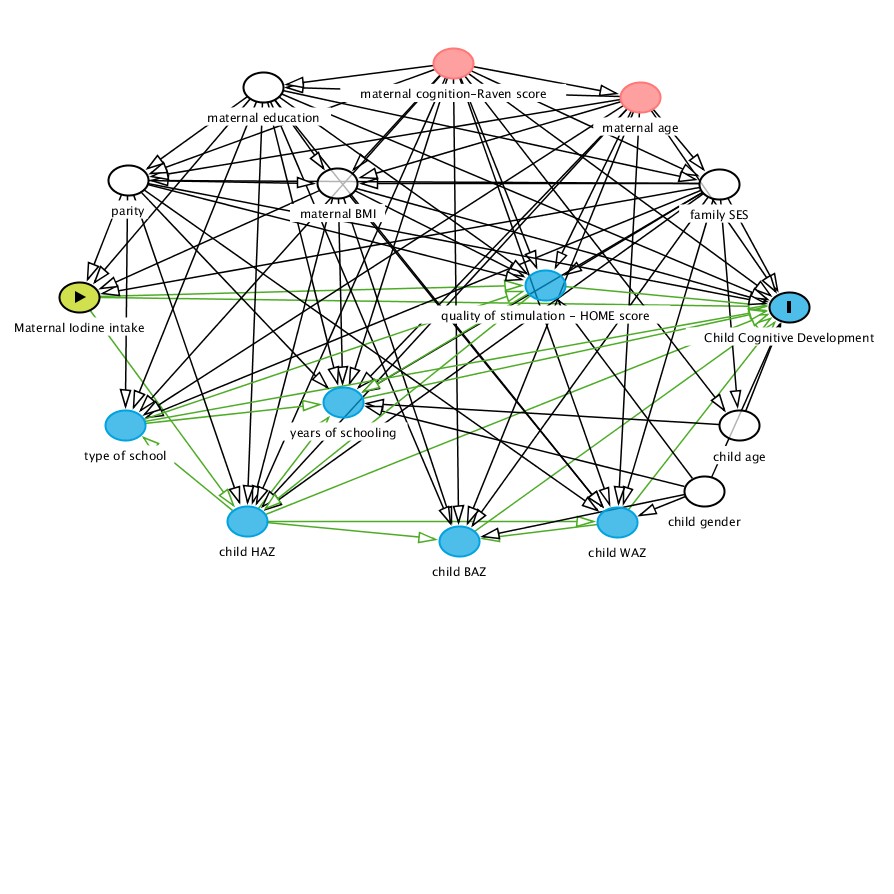


**Supplementary Figure S1.** Directed acyclic graph of the association between maternal iodine intake and child cognitive development. Blue nodes: variables associated with the outcome; pink nodes: confounders; white nodes: variables included in the minimal sufficient adjustment set for estimating the association of exposure with the outcome. The set contains child age and child gender as a priori selected adjustments and family socioeconomic status, maternal body mass index (BMI), maternal education, and parity as suggested by the DAG structure.


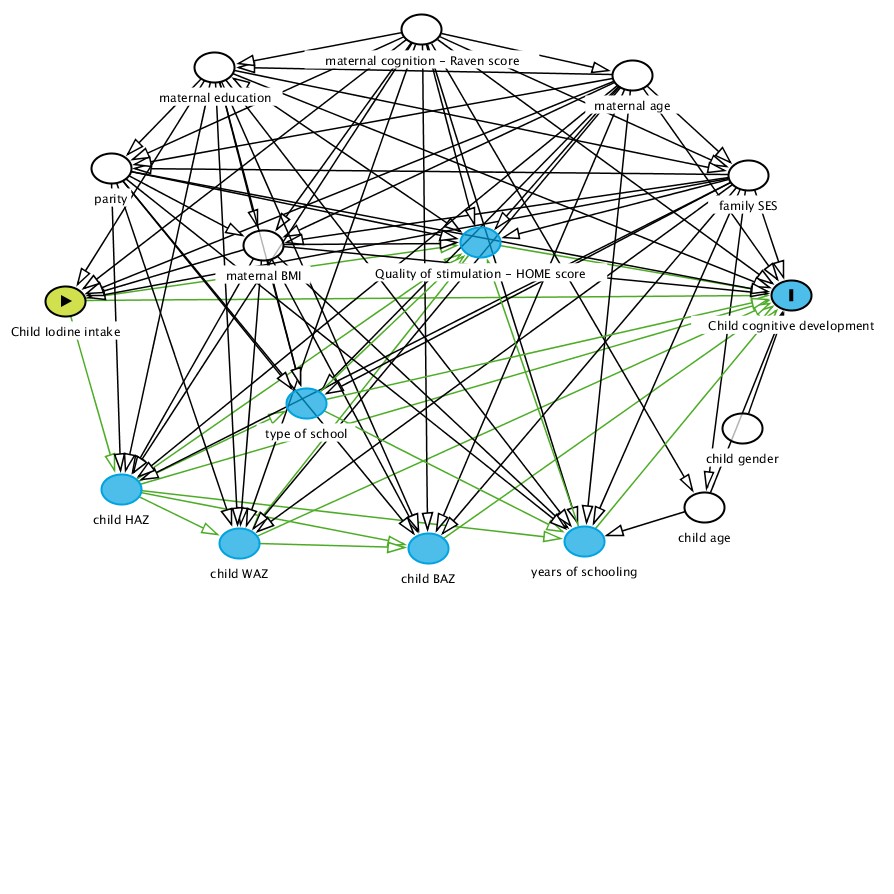


**Supplementary Figure S2.** Directed acyclic graph of the association between child iodine intake and cognitive development. Blue nodes: variables associated with the outcome; white nodes: variables included in the minimal sufficient adjustment set for estimating the association of exposure with the outcome. The set contains child age and child gender as a priori selected adjustments and family socioeconomic status, maternal body mass index (BMI), maternal age, maternal cognition (Raven score), maternal education, and parity as suggested by the DAG structure.

**Supplementary Figure S3.** Generalized additive models (GAMs) of maternal urinary iodine concentration (UIC) at gestational week 8 (GW8), adjusted for specific gravity and log_2_-transformed, with Wechsler Preschool & Primary Scale of Intelligence (WPPSI-III: full-scale, verbal scale, and performance scale); GAMs are adjusted for child age (continuous; years), child gender (categorical; male/female), and the tester of the assessment (categorical; 3 categories), family socioeconomic status (continuous; assets score), maternal body mass index (BMI; continuous; kg/m^2^), maternal education (continuous; years), and parity (continuous; number of children).

**Supplementary Figure S4.** Generalized additive models (GAMs) of maternal urinary iodine concentration (UIC) at gestational week 8 (GW8), adjusted for specific gravity and log_2_-transformed, with Wechsler Intelligence Scale for Children, Fourth Edition (WISC-IV: full-scale, verbal comprehension scale, perceptual reasoning scale, working memory scale, and processing speed scale); GAMs are adjusted for child age (continuous; years), child gender (categorical; male/female), and the tester of the assessment (categorical; 4 categories), family socioeconomic status (continuous; assets score), maternal body mass index (BMI; continuous; kg/m^2^), maternal education (continuous; years), and parity (continuous; number of children).

**Supplementary Figure S5.** Generalized additive models (GAMs) of child urinary iodine concentration (UIC) at 5 years, adjusted for specific gravity and log_2_-transformed, with Wechsler Preschool & Primary Scale of Intelligence (WPPSI-III: full-scale, verbal scale, and performance scale; GAMs are adjusted for child age (continuous; years), child gender (categorical; male/female), the tester of the assessment (categorical; 3 categories), family socioeconomic status (continuous; assets score), maternal body mass index (BMI; continuous; kg/m^2^), maternal age (continuous; years), maternal score at Raven’s test (continuous; raw score), maternal education (continuous; years), and parity (continuous; number of children).

**Supplementary Figure S6.** Generalized additive models (GAMs) of child urinary iodine concentration (UIC) at 5 years, adjusted for specific gravity and log_2_-transformed, with Wechsler Intelligence Scale for Children, Fourth Edition (WISC-IV: full-scale, verbal comprehension scale, perceptual reasoning scale, working memory scale, and processing speed scale); GAMs are adjusted for child age (continuous; years), child gender (categorical; male/female), the tester of the assessment (categorical; 4 categories), family socioeconomic status (continuous; assets score), maternal body mass index (BMI; continuous; kg/m^2^), maternal age (continuous; years maternal score at Raven’s test (continuous; raw score), maternal education (continuous; years), and parity (continuous; number of children).


**Supplementary Figure S7.** Generalized additive models (GAMs) of child urinary iodine concentration (UIC) at 10 years, adjusted for specific gravity and log_2_-transformed, with Wechsler Intelligence Scale for Children, Fourth Edition (WISC-IV: full-scale, verbal comprehension scale, perceptual reasoning scale, working memory scale, and processing speed scale); GAMs are adjusted for child age (continuous; years), child gender (categorical; male/female), the tester of the assessment (categorical; 4 categories), family socioeconomic status (continuous; assets score), maternal body mass index (BMI; continuous; kg/m^2^), maternal age (continuous; years), maternal score at Raven’s test (continuous; raw score), maternal education (continuous; years), and parity (continuous; number of children).

**Supplementary Table S1.** Comparison of maternal background characteristics and cognitive outcomes’ scores between participants with and without available

data on iodine intake [Urinary Iodine Intake (UIC) at pregnancy, 5, and 10 years of age)

|  | UIC at GW 8 – data available | | | UIC at 5y – data available | | | UIC at 10y – data available | | |
| --- | --- | --- | --- | --- | --- | --- | --- | --- | --- |
|  | Yes  (n = 1052) | No  (n = 478) |  | Yes  (n = 1156) | No  (n = 374) |  | Yes  (n = 1517) | No  (n = 13) |  |
|  | Mean (SD) | Mean (SD) | *P-value*^a^ | Mean (SD) | Mean (SD) | *P-value*^a^ | Mean (SD) | Mean (SD) | *P-value*^a^ |
| Age (years) | 26.7 (6.0) | 26.2 (6.0) | 0.109 | 26.7 (6.0) | 26.1 (6.0) | 0.072 | 26.5 (6.0) | 29.6 (7.2) | 0.127 |
| BMI (kg/m^2^) | 19.9 (2.5) | 20.3 (2.7) | 0.008 | 20.0 (2.5) | 20.3 (2.7) | 0.093 | 20.0 (2.6) | 20.4 (1.9) | 0.426 |
| Parity (continuous) | 1.5 (1.4) | 1.3 (1.3) | 0.012 | 1.5 (1.4) | 1.3 (1.4) | 0.037 | 1.4 (1.4) | 2.3 (2.0) | 0.086 |
| Family SES (assets score) | -0.3 (2.3) | 0.0 (2.2) | 0.013 | -0.2 (2.3) | -0.0 (2.2) | 0.229 | -0.2 (2.3) | -1.1 (2.5) | 0.180 |
| Education at 5y (years) | 4.4 (4.0) | 5.0 (3.9) | 0.006 | 4.5 (4.0) | 4.9 (4.0) | 0.083 | 4.6 (4.0) | 3.9 (4.6) | 0.603 |
| Education at 10y (years) | 5.0 (3.7) | 5.5 (3.7) | 0.004 | 5.1 (3.7) | 5.4 (3.7) | 0.101 | 5.1 (3.7) | 4.6 (3.9) | 0.688 |
| Raven’s score (continuous) | 23.6 (11.4) | 28.1 (11.6) | 0.000 | 24.0 (11.5) | 28.1 (11.6) | 0.000 | 25.0 (11.7) | 26.1 (12.4) | 0.840 |
| Full-scale-5y | 79.5 (23.6) | 78.7 (19.8) | 0.811 | 79.4 (23.3) | 78.5 (19.7) | 0.916 | - | - | - |
| Verbal-5y | 33.1 (11.4) | 32.2 (9.1) | 0.388 | 33.0 (11.1) | 32.4 (9.5) | 0.456 | - | - | - |
| Performance-5y | 34.2 (8.2) | 34.1 (7.1) | 0.794 | 34.3 (8.1) | 33.7 (7.1) | 0.334 | - | - | - |
| Full-scale*-*10y | 130.6 (33.6) | 136.3 (31.6) | 0.000 | 136.9 (31.4) | 130.9 (33.5) | 0.001 | 132.5 (33.2) | 115.4 (19.4) | 0.024 |
| Verbal comprehension*-*10y | 36.2 (10.7) | 38.0 (9.9) | 0.000 | 36.3 (10.7) | 38.0 (10.0) | 0.005 | 36.7 (10.6) | 32.7 (4.6) | 0.125 |
| Perceptual reasoning*-*10y | 31.1 (11.7) | 33.2 (11.0) | 0.000 | 31.2 (11.7) | 33.3 (11.1) | 0.001 | 29.8 (6.1) | 26.8 (5.0) | 0.294 |
| Working memory score*-*10y | 29.6 (6.2) | 30.0 (6.1) | 0.222 | 29.6 (6.2) | 30.1 (6.0) | 0.263 | 29.8 (6.1) | 26.1 (5.1) | 0.053 |
| Processing speed score*-*10y | 33.8 (11.7) | 35.1 (11.6) | 0.058 | 33.8 (11.8) | 35.5 (11.4) | 0.015 | 34.2 (11.7) | 27.9 (8.5) | 0.024 |

Abbreviations: UIC, urinary iodine concentration; GW, gestational week; BMI, Body mass index; SES, Socioeconomic status; 5y: 5 years; 10y: 10 years

^a^ *P*-values are based on Mann-Whitney U tests

**Supplementary Table S2.** References values and obtained concentrations of iodine in all reference materials included in the ICP-MS analyses for quality control

| Samples | n | Reference material | Reference value (mean±SD) or acceptable range | Obtained value (mean±SD) |
| --- | --- | --- | --- | --- |
| Maternal urine GW 8 (µg/L) | 109 | Seronorm Trace Elements Urine Blank, REF 201305, LOT OK4636 | 139±8 | 127±10 |
|  | 157 | Seronorm Trace Elements Urine, REF 201205, LOT NO2525 | 282±18 | 272±22 |
| Child urine 5y (µg/L) | 54 | Seronorm Trace Element Urine L-1, REF 210605, LOT 1011644 | 72-96 | 88±5 |
|  | 52 | Seronorm Trace Element Urine L-2, REF 210705, LOT 1011645 | 260-348 | 314±15 |
| Child urine 10y (µg/L) | 49 | Seronorm Trace Element Urine L-1, REF 210605, LOT 1011644 | 72-96 | 75±5 |
|  | 49 | Seronorm Trace Element Urine L-2, REF 210705, LOT 1011645 | 260-348 | 263±16 |

Abbreviations: SD, standard deviation; GW 8, gestational week 8; y, years.

**Supplementary Table S3.** Multivariable linear regression models of maternal urinary iodine concentrations at gestational week 8, categorized according to World Health Organization (2013) cut-offs for population iodine-intake classification, with their children’s cognitive abilities scores at 5 and 10 years of age, sensitivity analyses

|  | Categories of maternal urinary iodine (μg/L) at gestational week 8 | | | | | | | |
| --- | --- | --- | --- | --- | --- | --- | --- | --- |
|  | UIC < 150μg/L*^a^* | | | 150μg/L ≤ UIC < 500μg/L *^a^* | | UIC ≥ 500 μg/L*^a^* | | |
|  | n | B (95% CI) | *P-value* | n | Reference | n | B (95% CI) | *P-value* |
| Cognition at 5 years: *WPPSI-III ^b^* | | | | | | | | |
| Full-scale score | | | | | | | | |
| Model 1 | 259 | -1.6 (-4.6, 1.4) | 0.300 | 449 | Ref | 269 | -1.0 (-4.0, 2.1) | 0.538 |
| Model 2 | 277 | -1.3 (-4.3, 1.7) | 0.392 | 496 | Ref | 279 | -1.1 (-4.0, 1.9) | 0.482 |
| Verbal scale score | | | | | | | | |
| Model 1 | 259 | -1.1 (-2.7, 0.4) | 0.147 | 449 | Ref | 269 | -0.9 (-2.5, 0.7) | 0.259 |
| Model 2 | 277 | -1.2 (-2.7, 0.3) | 0.124 | 496 | Ref | 279 | -1.0 (-2.5, 0.5) | 0.187 |
| Performance scale score | | | | | | | | |
| Model 1 | 259 | -0.2 (-1.3, 1.0) | 0.771 | 449 | Ref | 269 | -0.2 (-1.3, 0.9) | 0.731 |
| Model 2 | 277 | 0.0 (-1.1, 1.1) | 0.950 | 496 | Ref | 279 | -0.1 (-1.2, 1.0) | 0.850 |
| Cognition at 10 years: *WISC IV ^c^* | | | | | | | | |
| Full-scale score |  |  |  |  |  |  |  |  |
| Model 1 | 259 | 0.7 (-3.6, 5.1) | 0.749 | 449 | Ref | 269 | -0.0 (-4.3, 4.3) | 0.992 |
| Model 2 | 277 | 0.2 (-4.0, 4.4) | 0.929 | 496 | Ref | 279 | -0.1 (-4.3, 4.0) | 0.946 |
| Verbal comprehension score | | | | | | | | |
| Model 1 | 259 | -0.7 (-2.1, 0.6) | 0.290 | 449 | Ref | 269 | -0.5 (-2.0, 0.9) | 0.458 |
| Model 2 | 277 | -0.6 (-1.9, 0.7) | 0.367 | 496 | Ref | 279 | -0.6 (-2.0, 0.8) | 0.394 |
| Perceptual reasoning score | | | | | | | | |
| Model 1 | 259 | 0.6 (-1.0, 2.2) | 0.474 | 449 | Ref | 269 | 0.4 (-1.2, 2.0) | 0.651 |
| Model 2 | 277 | 0.3 (-1.2, 1.8) | 0.700 | 496 | Ref | 279 | 0.3 (-1.3, 1.9) | 0.704 |
| Working memory score | | | | | | | | |
| Model 1 | 259 | 0.3 (-0.6, 1.2) | 0.481 | 449 | Ref | 269 | 0.5 (-0.4, 1.3) | 0.289 |
| Model 2 | 277 | 0.2 (-0.7, 1.0) | 0.683 | 496 | Ref | 279 | 0.4 (-0.4, 1.3) | 0.301 |
| Processing speed score | | | | | | | | |
| Model 1 | 259 | 0.5 (-1.1, 2.2) | 0.520 | 449 | Ref | 269 | -0.3 (-1.9, 1.3) | 0.697 |
| Model 2 | 277 | 0.3 (-1.3, 1.9) | 0.704 | 496 | Ref | 279 | -0.3 (-1.9, 1.3) | 0.713 |

Abbreviations: UIC, urinary iodine concentration; WPPSI-III, Wechsler Preschool & Primary Scale of Intelligence, Third Edition; WISC-IV, Wechsler Intelligence Scale for Children, Fourth Edition

*^a^* UIC is adjusted for specific gravity

*^b^* Analyses of outcomes at 5 years are adjusted for the full set of potential confounders included in the main analyses [child gender (categorical; male/female), child age (continuous; years), the tester of the assessment (categorical; 3 categories), maternal body mass index at enrolment (continuous; kg/m^2^), parity (continuous; number of children), family socioeconomic status at enrolment (continuous; assets score), maternal education at 5 years, (continuous; years); maternal age (continuous; years), maternal score at Raven’s test (continuous; raw score); Model 1 is additionally adjusted for maternal erythrocyte concentration levels of selenium, cadmium, and arsenic at GW14; Model 2 is additionally adjusted for the multiple micronutrient supplementation group during pregnancy (Fe30mg & Fol400μg or Fe 60mg & Fol400μg or Fe30mg & Fol400μg & 13 additional micronutrients including 150 μg of iodine).

*^c^* Analyses of outcomes at 10 years are adjusted for the full set of potential confounders included in the main analyses [child gender (categorical; male/female), child age (continuous; years), the tester of the assessment (categorical; 4 categories), maternal body mass index at enrolment (continuous; kg/m^2^), parity (continuous; number of children), family socioeconomic status at enrolment (continuous; assets score), maternal education at 10 years (continuous; years); maternal age (continuous; years), maternal score at Raven’s test (continuous; raw score); Model 1 is additionally adjusted for maternal erythrocyte concentration levels of selenium, cadmium, and arsenic at GW14; Model 2 is additionally adjusted for the multiple micronutrient supplementation group during pregnancy (Fe30mg & Fol400μg or Fe 60mg & Fol400μg or Fe30mg & Fol400μg & 13 additional micronutrients including 150 μg of iodine)

**Supplementary Table S4.** Multivariable linear regression models of child urinary iodine concentrations at 5 years (categorized according to World Health Organization (2013) cut-offs for population iodine-intake classification) with cognitive abilities’ scores children at 5 and 10 years of age, sensitivity analyses

|  | Categories of child urinary iodine (μg/L) at 5 years | | | | | | | |
| --- | --- | --- | --- | --- | --- | --- | --- | --- |
|  | UIC < 100μg/L *^a^* | | | 100μg/L ≤ UIC < 300μg/L *^a^* | | UIC ≥ 300 μg/L *^a^* | | |
|  | n | B (95% CI) | *P-value* | n | Reference | n | B (95% CI) | *P-value* |
| Cognition at 5 years: *WPPSI-III ^b^* | | | | | | | | |
| Full-scale score |  |  |  |  |  |  |  |  |
| Model 1 | 43 | -0.3 (-6.2, 5.5) | 0.912 | 327 | Ref | 786 | **2.7 (0.2, 5.3)** | 0.035 |
| Model 2 | 43 | -0.5 (-6.5, 5.4) | 0.857 | 327 | Ref | 786 | **2.9 (0.3, 5.4)** | 0.027 |
| Verbal scale score |  |  |  |  |  |  |  |  |
| Model 1 | 43 | 1.2 (-1.4, 3.8) | 0.355 | 327 | Ref | 786 | 1.0 (-0.2, 2.3) | 0.111 |
| Model 2 | 43 | 1.2 (-1.4, 3.8) | 0.373 | 327 | Ref | 786 | 1.1 (-0.2, 2.4) | 0.097 |
| Performance scale score |  |  |  |  |  |  |  |  |
| Model 1 | 43 | -1.7 (-3.9, 0.5) | 0.129 | 327 | Ref | 786 | 0.9 (-0.0, 1.8) | 0.063 |
| Model 2 | 43 | -1.8 (-4.0, 0.5) | 0.121 | 327 | Ref | 786 | 0.8 (-0.1, 1.8) | 0.073 |
| Cognition at 10 years: *WISC IV ^c^* | | | | | | | | |
| Full-scale score | | | | | | | | |
| Model 1 | 43 | -4.4 (-11.5, 2.6) | 0.219 | 327 | Ref | 786 | 1.2 (-2.7, 5.0) | 0.553 |
| Model 2 | 43 | -5.0 (-12.1, 2.2) | 0.172 | 327 | Ref | 786 | 1.6 (-2.2, 5.3) | 0.415 |
| Verbal comprehension score | | | | | | | | |
| Model 1 | 43 | 0.4 (-1.9, 2.6) | 0.752 | 327 | Ref | 786 | 0.4 (-0.8, 1.5) | 0.525 |
| Model 2 | 43 | 0.2 (-2.0, 2.5) | 0.834 | 327 | Ref | 786 | 0.5 (-0.7, 1.6) | 0.413 |
| Perceptual reasoning score | | | | | | | | |
| Model 1 | 43 | -1.7 (-4.3, 1.0) | 0.213 | 327 | Ref | 786 | 0.2 (-1.2, 1.6) | 0.751 |
| Model 2 | 43 | -1.9 (-4.6, 0.7) | 0.158 | 327 | Ref | 786 | 0.5 (-0.9, 1.8) | 0.512 |
| Working memory score | | | | | | | | |
| Model 1 | 43 | -0.1 (-1.9, 1.6) | 0.864 | 327 | Ref | 786 | 0.5 (-0.3, 1.3) | 0.232 |
| Model 2 | 43 | -0.2 (-1.9, 1.5) | 0.833 | 327 | Ref | 786 | 0.5 (-0.2, 1.3) | 0.166 |
| Processing speed score | | | | | | | | |
| Model 1 | 43 | -3.0 (-6.0, 0.0) | 0.053 | 327 | Ref | 786 | 0.1 (-1.3, 1.5) | 0.912 |
| Model 2 | 43 | -3.1 (-6.1, 0.2) | 0.039 | 327 | Ref | 786 | 0.1 (-1.3, 1.5) | 0.899 |

Abbreviations: UIC, urinary iodine concentration; WPPSI-III, Wechsler Preschool & Primary Scale of Intelligence, Third Edition; WISC-IV, Wechsler Intelligence Scale for Children, Fourth Edition

*^a^* UIC is adjusted for specific gravity

*^b^* Analyses of outcomes at 5 years are adjusted for the full set of potential confounders included in the main analyses [child gender (categorical; male/female), child age (continuous; years), the tester of the assessment (categorical; 3 categories), maternal body mass index at enrolment (continuous; kg/m^2^), parity (continuous; number of children), family socioeconomic status at enrolment (continuous; assets score), maternal education at 5 years, (continuous; years); maternal age (continuous; years), maternal score at Raven’s test (continuous; raw score); Model 1 is additionally adjusted for maternal erythrocyte concentration levels of selenium, cadmium, and arsenic at GW14; Model 2 is additionally adjusted for the multiple micronutrient supplementation group during pregnancy (Fe30mg & Fol400μg or Fe 60mg & Fol400μg or Fe30mg & Fol400μg & 13 additional micronutrients including 150 μg of iodine).

*^c^* Analyses of outcomes at 10 years are adjusted for the full set of potential confounders included in the main analyses [child gender (categorical; male/female), child age (continuous; years), the tester of the assessment (categorical; 4 categories), maternal body mass index at enrolment (continuous; kg/m^2^), parity (continuous; number of children), family socioeconomic status at enrolment (continuous; assets score), maternal education at 10 years (continuous; years), maternal age (continuous; years), maternal score at Raven’s test (continuous; raw score); Model 1 is additionally adjusted for maternal erythrocyte concentration levels of selenium, cadmium, and arsenic at GW14; Model 2 is additionally adjusted for the multiple micronutrient supplementation group during pregnancy (Fe30mg & Fol400μg or Fe 60mg & Fol400μg or Fe30mg & Fol400μg & 13 additional micronutrients including 150 μg of iodine)

**Supplementary Table S5.** Multivariable linear regression models of child urinary iodine concentrations at 10 years (categorized according to World Health Organization (2013) cut-offs for population iodine-intake classification) with cognitive abilities’ scores children at 10 years of age, sensitivity analyses

|  | Categories of child urinary iodine (μg/L) at 10 years | | | | | | | |
| --- | --- | --- | --- | --- | --- | --- | --- | --- |
|  | UIC < 100μg/L *^a^* | | | 100μg/L ≤ UIC < 300μg/L *^a^* | | UIC ≥ 300 μg/L *^a^* | | |
|  | n | B (95% CI) | *P-value* | n | Reference | n | B (95% CI) | *P-value* |
| Cognition at 10 years: *WISC IV* *^b^* | | | | | | | | |
| Full-scale score | | | | | | | | |
| Model 1 | 59 | -0.3 (-8.1, 7.5) | 0.939 | 712 | Ref | 746 | -1.5 (-4.4, 1.4) | 0.316 |
| Model 2 | 59 | -0.3 (-8.2, 7.6) | 0.945 | 712 | Ref | 746 | -1.4 (-4.4, 1.5) | 0.336 |
| Verbal comprehension score | | | | | | | | |
| Model 1 | 59 | 1.9 (-0.4, 4.2) | 0.108 | 712 | Ref | 746 | -0.3 (-1.3, 0.6) | 0.503 |
| Model 2 | 59 | 1.8 (-0.5, 4.2) | 0.129 | 712 | Ref | 746 | -0.3 (-1.2, 0.7) | 0.599 |
| Perceptual reasoning score | | | | | | | | |
| Model 1 | 59 | 0.2 (-2.9, 3.4) | 0.883 | 712 | Ref | 746 | -0.7 (-1.7, 0.4) | 0.211 |
| Model 2 | 59 | 0.2 (-2.9, 3.4) | 0.883 | 712 | Ref | 746 | -0.7 (-1.7, 0.4) | 0.220 |
| Working memory score | | | | | | | | |
| Model 1 | 59 | 0.3 (-1.0, 1.7) | 0.624 | 712 | Ref | 746 | -0.0 (-0.6, 0.6) | 0.942 |
| Model 2 | 59 | 0.4 (-1.0, 1.7) | 0.582 | 712 | Ref | 746 | -0.0 (-0.6, 0.6) | 0.908 |
| Processing speed score | | | | | | | | |
| Model 1 | 59 | **-2.8 (-5.3, -0.2)** | 0.035 | 712 | Ref | 746 | -0.5 (-1.6, 0.6) | 0.396 |
| Model 2 | 59 | **-2.7 (-5.3, -0.1)** | 0.040 | 712 | Ref | 746 | -0.5 (-1.6, 0.6) | 0.384 |

Abbreviations: UIC, urinary iodine concentration; WISC-IV, Wechsler Intelligence Scale for Children, Fourth Edition

*^a^* UIC is adjusted for specific gravity

*^b c^* Analyses of outcomes at 10 years are adjusted for the full set of potential confounders included in the main analyses [child gender (categorical; male/female), child age (continuous; years), the tester of the assessment (categorical; 4 categories), maternal body mass index at enrolment (continuous; kg/m^2^), parity (continuous; number of children), family socioeconomic status at enrolment (continuous; assets score), maternal education at 10 years (continuous; years), maternal age (continuous; years), maternal score at Raven’s test (continuous; raw score); Model 1 is additionally adjusted for maternal erythrocyte concentration levels of selenium, cadmium, and arsenic at GW14; Model 2 is additionally adjusted for the multiple micronutrient supplementation group during pregnancy (Fe30mg & Fol400μg or Fe 60mg & Fol400μg or Fe30mg & Fol400μg & 13 additional micronutrients including 150 μg of iodine)
